# Cluster Headache Secondary to Macroprolactinoma with Ipsilateral Cavernous Sinus Invasion

**DOI:** 10.1155/2012/830469

**Published:** 2012-09-23

**Authors:** M. J. Levy, I. Robertson, T. A. Howlett

**Affiliations:** ^1^Department of Endocrinology, Leicester Royal Infirmary, Leicester LE1 5WW, UK; ^2^Department of Neurosurgery, Queens Medical Centre, Nottingham NG7 2UH, UK

## Abstract

We present a 25 year-old man with episodic cluster headache that was refractory to all standard pharmacological prophylactic and abortive treatments. Because of the lack of response, an MRI brain was performed which showed a large pituitary tumour with ipsilateral cavernous sinus invasion. The serum prolactin was significantly elevated at 54,700 miU/L (50–400) confirming a macro-prolactinoma. Within a few days of cabergoline therapy the headache resolved. He continues to be headache free several years after starting the dopamine agonist. This case highlights the importance of imaging the pituitary fossa in patients with refractory cluster headache, It also raises the potential anatomical importance of the cavernous sinus in pituitary-associated headache.

## 1. Case Presentation

A 25-year-old man presented with increasingly severe right-sided headache attacks with ipsilateral cranial autonomic features. His first attack was characterised by a sharp retroorbital pain with ipsilateral lacrimation, conjunctival congestion, and nasal stuffiness. He was agitated throughout the pain, which lasted 15–30 minutes, and he slept for seven hours afterwards. Three months later he experienced an identical attack, and the frequency gradually increased to between two and three episodes per day. The attacks were stereotypical, being exclusively right-sided with extreme agitation during the pain. Prior to seeking medical help, he was contemplating suicide due to the severity of his symptoms. He had previously been fit and well apart from a history of migraine. The latter was described as intermittent mild-to-moderate headache attacks lasting several hours with associated photophobia, phonophobia, and aggravation with movement, easily distinguishable from the current headache presentation.

**Figure 1:**
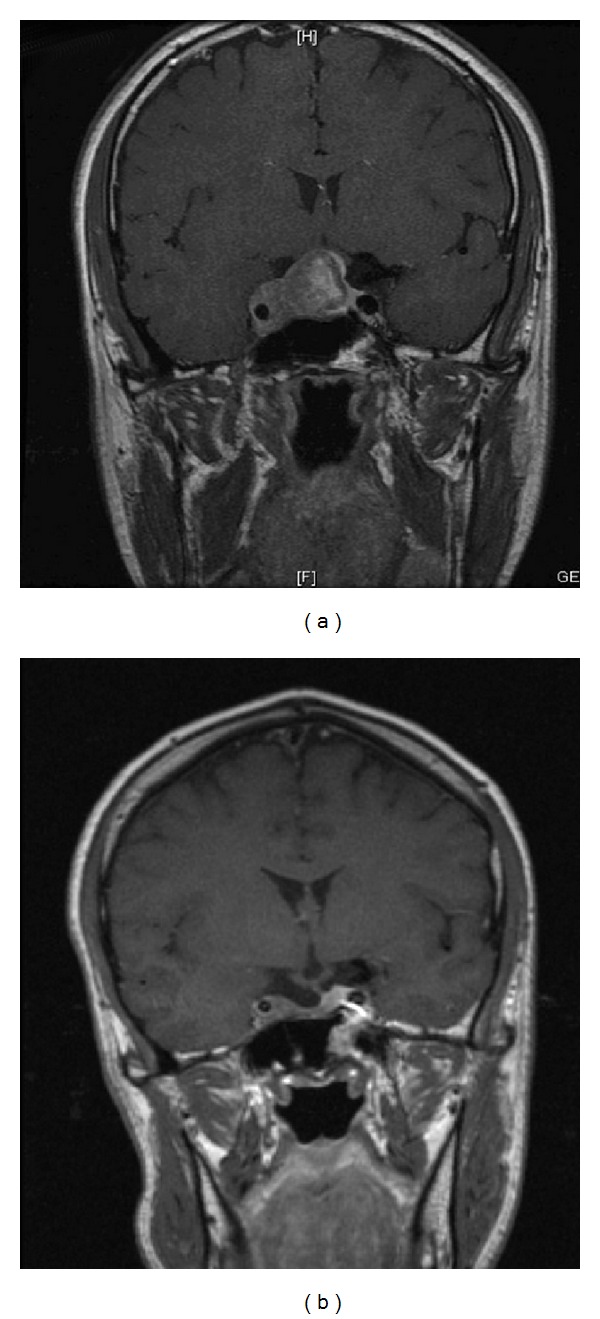
Pretreatment and postcabergoline MRI showing resolution of right-sided cavernous sinus invasion.

A diagnosis of cluster headache was made and abortive therapy with subcutaneous sumatriptan and high-flow oxygen was given, with no clinical response. Prophylactic therapy with high-dose verapamil did not have any effect on the attack frequency or duration. Given the refractory symptoms, an MRI scan was performed which showed a large pituitary adenoma with right-sided cavernous sinus invasion (Figure 1). The serum prolactin was significantly elevated at 54,700 mU/L (normal range 50–400 mU/L), with normal IGF1 and growth hormone 0.4 mU/L, excluding acromegaly; the remainder of the pituitary function was normal. A pituitary macroprolactinoma was diagnosed and the patient was prescribed cabergoline 0.5 mg twice/week. Over the next four days he described a sensation of parasthesia over the scalp, since which he has not experienced a single headache attack. An MRI scan performed several months later showed significant reduction in tumour volume with resolution of cavernous sinus invasion (Figure 1). Since followup in the endocrine clinic, he has become gonadotropin deficient, requiring testosterone gel replacement, and he remains headache-free with a serum prolactin of 627 mU/L.

## 2. Discussion

This case of a macroprolactinoma presenting with refractory cluster headache showed complete resolution of symptoms within days of starting dopamine agonist treatment. The close temporal relationship between cabergoline and cessation of headache suggests that the pituitary lesion was the direct cause of the cluster headache. The probable mechanism is direct irritation of the trigeminal nerve within the cavernous sinus, although previous investigation of pituitary tumour-associated headache has shown no relationship between cavernous sinus invasion or tumour volume and headache [1]. Pituitary lesions have been reported with the range of autonomic headache syndromes (trigeminal autonomic cephalgias), including cluster headache, SUNCT, paroxysmal hemicranias, and hemicrania continua. This has led to an interest in the link between pituitary tumours and headache [2]. Prolactinomas may be particularly associated with headache and there are previous reports of both cluster headache and SUNCT associated with ipsilateral cavernous sinus invasion as in this patient, with resolution of headache after administration of dopamine agonist therapy and reversal of cavernous sinus invasion due to tumour shrinkage [3–6]. Interestingly, there are also reports of SUNCT exacerbations after the administration of cabergoline [7, 8], suggesting that the dopamine-prolactin axis is important in the pathophysiology of this headache syndrome.

In primary cluster headache and SUNCT, the relative importance of central nervous and cavernous activation has been debated, and the demonstration of ipsilateral hypothalamic activation during an attack is more suggestive of a central nervous disorder than a primary problem of the cavernous sinus [9, 10]. It is not uncommon for patients with macroprolactinoma to have extensive cavernous sinus invasion, and the quick response to dopamine agonist therapy in terms of tumour mass reduction and normalisation of prolactin is typical for this condition. However, the presentation with cluster headache and rapid abolition of symptoms sets this case apart from the usual clinical features of macroprolactinoma.

This case highlights the importance of close collaboration between endocrinologists and headache specialists because pituitary tumours may present with severe headache. If the clinician images the pituitary fossa and finds the prolactin to be significantly elevated, then dopamine agonists may abort the headache and normalise the prolactin without the need for prophylactic headache medications. We would advocate close imaging of the pituitary fossa in patients presenting with atypical or refractory cluster headache, in case that symptoms are secondary to an underlying pituitary tumour.
